# Differential bone and vessel type formation at superior and dura periosteum during cranial bone defect repair

**DOI:** 10.1038/s41413-024-00379-9

**Published:** 2025-01-13

**Authors:** Yuankun Zhai, Zhuang Zhou, Xiaojie Xing, Mark Nuzzle, Xinping Zhang

**Affiliations:** Center for Musculoskeletal Research, University of Rochester, School of Medicine and Dentistry, Rochester, NY USA

**Keywords:** Dental diseases, Bone

## Abstract

The cranial mesenchyme, originating from both neural crest and mesoderm, imparts remarkable regional specificity and complexity to postnatal calvarial tissue. While the distinct embryonic origins of the superior and dura periosteum of the cranial parietal bone have been described, the extent of their respective contributions to bone and vessel formation during adult bone defect repair remains superficially explored. Utilizing transgenic mouse models in conjunction with high-resolution multiphoton laser scanning microscopy (MPLSM), we have separately evaluated bone and vessel formation in the superior and dura periosteum before and after injury, as well as following intermittent treatment of recombinant peptide of human parathyroid hormone (rhPTH), Teriparatide. Our results show that new bone formation along the dura surface is three times greater than that along the superior periosteal surface following injury, regardless of Teriparatide treatment. Targeted deletion of PTH receptor PTH1R via *SMA-CreER* and *Col 1a (2.3)-CreER* results in selective reduction of bone formation, suggesting different progenitor cell pools in the adult superior and dura periosteum. Consistently, analyses of microvasculature show higher vessel density and better organized arterial-venous vessel network associated with a 10-fold more osteoblast clusters at dura periosteum as compared to superior periosteum. Intermittent rhPTH treatment further enhances the arterial vessel ratio at dura periosteum and type H vessel formation in cortical bone marrow space. Taken together, our study demonstrates a site-dependent coordinated osteogenic and angiogenic response, which is determined by regional osteogenic progenitor pool as well as the coupling blood vessel network at the site of cranial defect repair.

## Introduction

Bone defects caused by traumatic injury, tumor resection, and various congenital and pathological bone losses are functionally debilitating and financially burdensome. Although a small-sized defect can heal spontaneously by itself, a large sized defect is often difficult to heal, leading to scar tissue formation and permanent disabilities. Cranioplasty is a common neurosurgical procedure performed to reconstruct the missing cranial bone segments to protect cerebral structures and to restore cerebrospinal fluid and blood flow.^1^ While autografts are commonly used, allografts (bony materials from cadavers) and synthetic materials are gaining favor owing to several benefits they offer, including reduced surgery time, potential for customized fitting, and elimination of donor site complications.^2^ However, both allograft and synthetic materials often exhibit poor osseointegration compared to autograft primarily due to the limited osteogenic and angiogenic activities of the skull bone, along with the compromised regenerative environment at the site of healing.

Teriparatide, the human recombinant parathyroid hormone peptides (rhPTH), has been used in combination with various bone graft materials for bone repair and reconstruction. It is currently approved in various clinical trials for bone repair and defect reconstruction.^3–6^ Teriparatide is a synthetic form of the natural human parathyroid hormone (PTH), consisting of the first 34 amino acids of the N-terminus of PTH. Intermittent administration of Teriparatide has been shown to exert an anabolic effect in animal models, leading to accelerated long-bone fracture healing and improved periosteal bone callus formation.^7^ Intermittent rhPTH treatment is further shown to enhance cranial bone defect healing and facilitates osseointegration of the cranial bone allografts.^8^ PTH acts through its G-protein coupled receptor, PTH/PTHrP type I receptor (PTH1R), which primarily utilizes the PKA pathway to modulate the mineral ion homeostasis and exert both catabolic and anabolic effects on bone tissue.^9,10^ More recent studies suggest that the anabolic effects of PTH could arise from activation of divergent pathways, including Wnt/β-catenin pathway.^11–14^

Neurocranium, also known as calvarium, consists of frontal, occipital, and parietal bones, forming a protective cavity for brain. The calvarial bone is enveloped by superior periosteum at its outer surface and dura periosteum at its inner surface. Both superior and dura periosteum are thin layers of connective tissue membranes firmly attached to the surface of bone.^15–17^ Distinct from long bones, adult calvarial bone has a mixed embryonic origin. Mouse frontal bone is derived from neural crest whereas the parietal bone is derived from paraxial mesoderm and the occipital bone is mixed in origin. In addition to the difference in the origin of the bones, suture and dura mater of the calvarium can also be traced to different origins, adding significant complexity and reginal specificity to the cranial bone tissue. Uniquely to the parietal bone, its periosteum is derived from mesoderm whereas the dura that covers the inner surface of parietal bone is derived from the neural crest.^18,19^ Both superior and dura periosteum are believed to contain multipotent progenitor cells that directly contribute to postnatal cranial bone tissue remodeling, repair, and regeneration.

While the superior and dura periosteum have been recognized for their pivotal roles in postnatal bone repair, their respective contributions and underlying mechanisms in defect repair have only been superficially investigated. Given the distinct embryonic origins of the superior and dura periosteum during development, it is crucial to explore the diverse pools of progenitor cells engaged in postnatal and adult repair and regeneration processes. In our current study, we examined the osteogenic and angiogenic activities of the superior and dura periosteum at the parietal bone of the calvarium during spontaneous and rhPTH-enhanced bone defect repair. Our data suggest that superior and dura periosteum harbors distinct osteoprogenitors that exhibit different osteogenic potential. By examination of the blood vessel type distribution in superior and dura periosteum utilizing high-resolution multiphoton laser scanning microscopy (MPLSM), our study further demonstrates a differential response that depends on an organized angiogenesis coupling to osteoblasts at superior and dura periosteum during calvarial bone defect repair. The study provides new insights into the mechanisms of calvarial bone healing, offering potential directions for repair and tissue engineering of bone tissue at the site of cranial bone defects.

## Results

### Differential contribution of superior and dura periosteum to spontaneous and rhPTH-treated bone defect repair

**Fig. 1:**
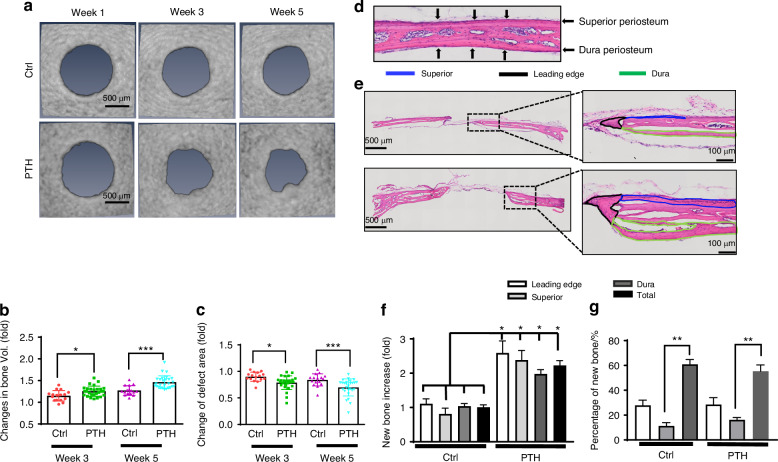
Differential new bone formation at the superior and dura periosteum in spontaneous and intermittent PTH-enhanced repair. **a** Longitudinal MicroCT images of cranial defect at weeks 1, 3, and 5 post-op. **b**, **c** Quantitative longitudinal MicroCT analyses to show fold of changes of the bone volume and defect area at weeks 3 and 5 as compared to week 1 post-op. **d** H&E staining of the superior and dura periosteum on parietal bone prior to injury. **e** H&E staining of the defect treated with or without rhPTH at 1X and 20X magnification, outlining the new bone formation at the leading edge (black), superior (blue), and dura periosteum (green) at the bone defects weeks 5 post-op. **f** Quantitative histomorphometric analyses to show changes of bone formation at the leading edge, superior, and dura periosteum with or without rhPTH treatment. **g** The percentage of new bone formation at the leading edge, superior periosteum, and dura periosteum as compared to the total bone at the defect site. *n* ≥ 16, *P < 0.05, **P < 0.01 by ANOVA

Longitudinal MicroCT analyses were performed at weeks 1, 3 and 5 following surgery to determine bone formation within or around bone defect. Progressively increased bone formation was noted at the leading edge of the defect as well as in the surrounding area in control defects and the defects treated with intermittent rhPTH (Fig. 1a). Quantitative analyses showed an average 1.15-fold of increase in new bone formation at week 3 and a 1.23-fold of increase at week 5 post-surgery in untreated defects. Intermittent rhPTH treatment increased the bone volume at the repair site by an average of 1.27-fold at week 3 and 1.43-fold at week 5 (Fig. 1b, *n* ≥ 16, *P* < 0.05). The rhPTH treatment further reduced the defect area by an average of 31% at week 5 as compared to a 16% decrease in the controls (Fig. 1c, n ≥ 16, *P* < 0.05). Histologic analyses showed that prior to injury, superior and dura periosteum were thin layers of tissue attached to the surface of the parietal bone (Fig. 1d). Following injury and treatment of rhPTH, new bone could be identified at the leading edge of the defect and along the outer (superior periosteal) and inner (dura periosteal) surface of the parietal bone (Fig. 1e, Fig. S1). Histomorphometric analyses revealed increased new bone formation at all three sites, with approximately 11% of new bone at the superior periosteal surface, 28% at the leading edge, and 61% along the dura periosteal surface (Fig. 1f & g, *n* ≥ 16, *P* < 0.01). While rhPTH significantly increased new bone formation at all three sites, approximately 3.4-fold more new bone was observed along the surface of dura compared to the surface of superior periosteum, suggesting a much stronger regenerative response at the dura periosteum than superior periosteum.

### Selective inhibition of bone formation at superior and dura periosteum following targeted deletion of PTH1R

**Fig. 2:**
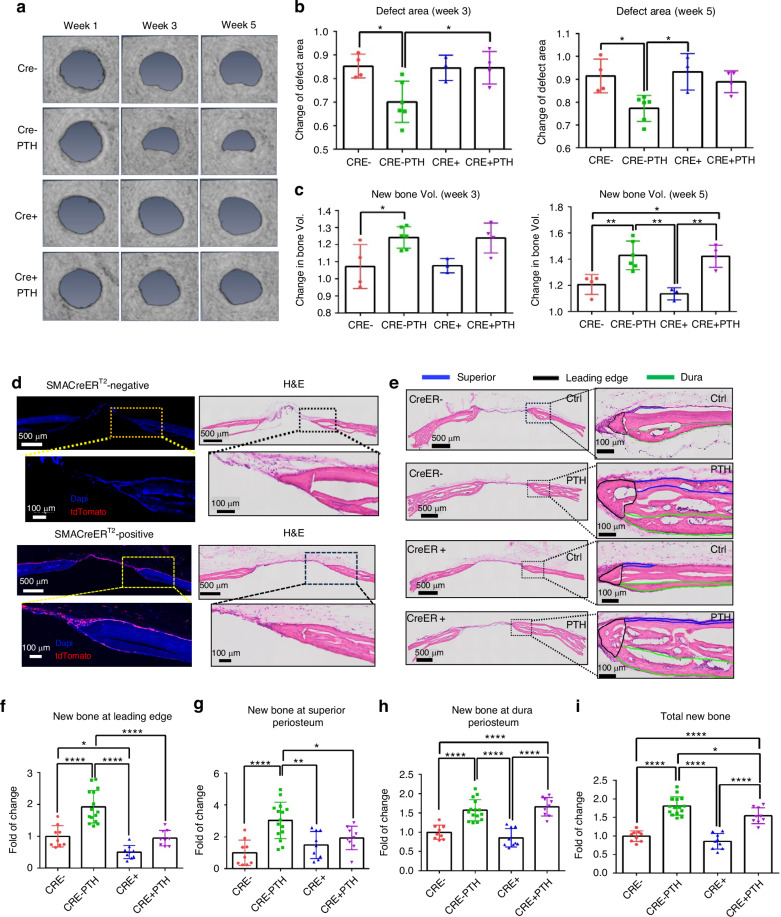
Impaired defect repair at superior periosteum and leading edge but not at dura periosteum following targeted deletion of *PTH1R* via SMA-CreER^T2^. **a** Longitudinal MicroCT images of the cranial defect in Cre^−^ and Cre^+^ mice treated with or without rhPTH at weeks 1, 3, and 5 post-op. Quantitative MicroCT analyses to show fold of changes of the defect area (**b**) and bone volume (**c**) at weeks 3 and 5. **d** Fluorescent images and the corresponding H&E histology of cranial defects, illustrating targeting cells in SMA-CreER^T2^; Ai9 mice following Tamoxifen injection. **e** Histologic H&E staining of the defect treated with or without rhPTH at 1X and 20X magnification, outlining the new bone formation at the leading edge (black), superior (blue), and dura periosteum (green) of the bone defects at week 5 post-surgery. Quantitative histomorphometric analyses to show changes of new bone formation at the leading edge (**f**), superior (**g**), dura periosteum (**h**), and all areas combined (**i**) with or without rhPTH treatment. *n* = 3–6, *P < 0.05, **P < 0.01, ****P < 0.000 1 by ANOVA

**Fig. 3:**
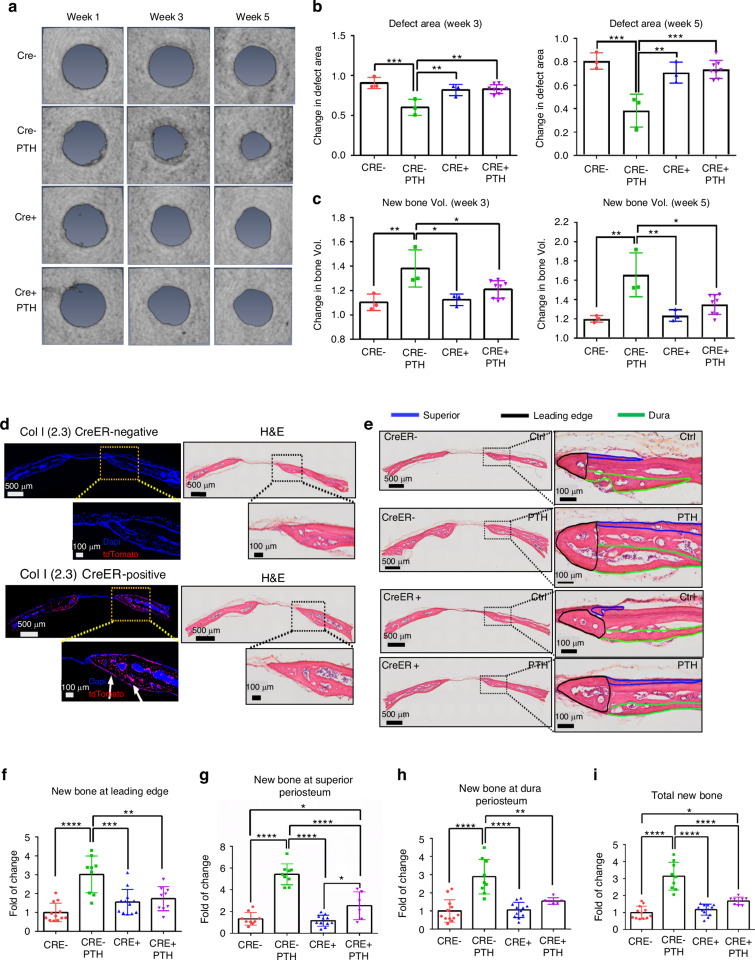
Impaired defect repair at all three sites following targeted deletion of *PTH1R* via Col 1a (2.3)-CreER^T2^. **a** Longitudinal MicroCT images of cranial defect in Cre^−^ and Cre^+^ mice treated with or without rhPTH at weeks 1, 3, and 5 post-op. Quantitative MicroCT analyses to show fold of changes of the defect area (**b**) and bone volume (**c**) at weeks 3 and 5. **d** Fluorescent images and the corresponding H&E histology of cranial defects, illustrating targeting cells in Col 1a (2.3)-CreER^T2^; Ai9 mice following Tamoxifen injection. **e** Histologic H&E staining of the defect treated with or without rhPTH at 1X and 20X magnification, outlining the new bone formation at the leading edge (black), superior (blue), and dura periosteum (green) of the bone defects at week 5 post-surgery. Quantitative histomorphometric analyses to show changes of new bone formation at the leading edge (**f**), superior (**g**), dura periosteum (**h**), and all areas combined (**i**) with or without rhPTH treatment. *n* = 4–7, *P < 0.05, **P < 0.01, ****P < 0.001 by ANOVA

To further characterize the different bone formation response at dura and superior periosteum, we examined the rhPTH-enhanced repair following CreER-mediated conditional deletion of PTH receptor, *PTH1R*, in periosteal mesenchymal progenitors via SMA-CreER^T2^ and osteoblasts via *Col 1a (2.3)-CreER*^*T2*^ mouse models. MicroCT longitudinal analyses showed a modest reduction in defect area at week 5 in *SMA-CreER*^*T2*^; *PTH1R*^*f/f*^ mice as compared to the controls, although this difference was not statistically significant (Fig. 2a–c, *P* > 0.05). Targeting efficiency of *SMA-CreER*^*T2*^ was subsequently examined by crossing the *SMA-CreER*^*T2*^ mice with Ai9 tdTomato reporter mice. As shown, *SMA-CreER*^*T2*^; Ai9 mice labeled superior periosteum, connective tissue within the defect, and the new bone formed at the leading edge of the cranial defect, with few cells labeled along dura periosteum (Fig. 2d). While no significant changes were seen in MicroCT analyses, careful examination of the defect healing via histologic analyses showed that targeted deletion of *PTH1R* via *SMA-CreER*^*T2*^ selectively reduced new bone formation around defect site at week 5 following surgery (Fig. 2e). Quantitative histomorphometric analyses showed a significant reduction of new bone formation on the superior periosteal surface and at the leading edge of bone defect (*P* < 0.05) with little effect observed along the dura surface following rhPTH treatment (Fig. 2f–i). The effects of targeted deletion of *PTH1R* via *Col 1a (2.3)-CreER*^*T2*^ were also examined at superior and dura periosteum. In contrast to *SMA-CreER*^*T2*^; *PTH1R*^*f/f*^ mice, deletion of *PTH1R* via *Col 1a (2.3)-CreER*^*T2*^ had a significant reduction of new bone formation at both weeks 3 and 5 following rhPTH treatment as demonstrated by MicroCT analyses (Fig. 3a, b, *P* < 0.05). Accordingly, the reduction of defect area following rhPTH treatment was also attenuated (Fig. 3c, *P* < 0.05). The analyses of *Col 1a (2.3)-CreER*^*T2*^; Ai9 reporter mice via fluorescence microscopy showed that *Col 1a (2.3)-CreER*^*T2*^ labeled all osteoblasts on superior, dura, leading edge of the defect, as well as in bone tissue (Fig. 3d). Further histomorphometric analyses confirmed the reduction of new bone in all three regions in rhPTH treated samples compare to the controls (Fig. 3f–i, *P* < 0.05).

### Differential distribution of blood vessel type and active osteoblast clusters in dura and superior periosteum

**Fig. 4:**
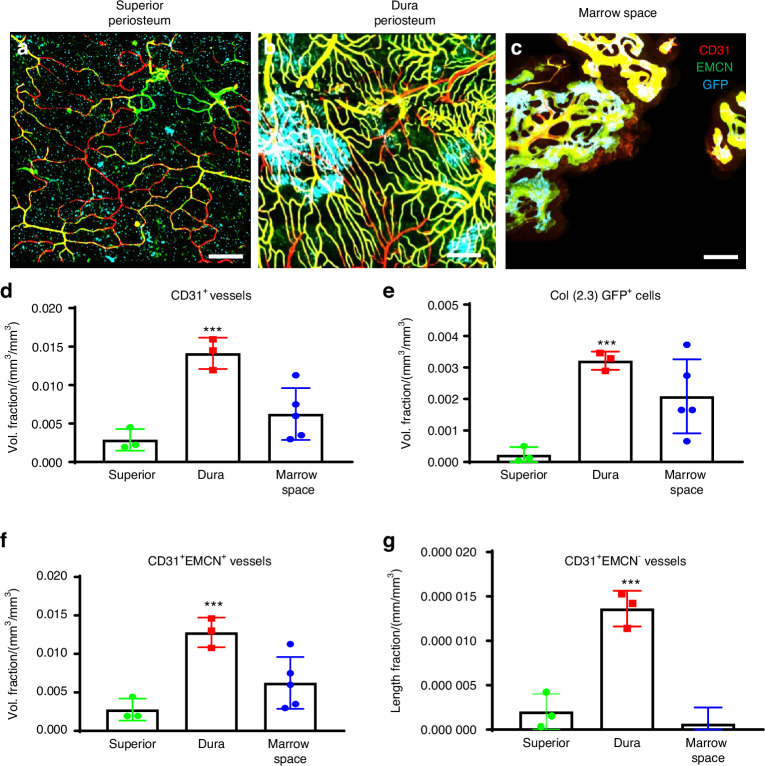
Superior periosteum had fewer vessels and active osteoblasts as compared to dura periosteum and cortical bone marrow space prior to injury. Representative MPLSM image of vessel network in superior periosteum (**a**), dura periosteum (**b**), and bone marrow space (**c**). Quantitative MPLSM analyses of microvasculature to show volume fractions of the total CD31^+^ vessels (**d**), Col 1a (2.3) GFP^+^ osteoblast volume (**e**), and CD31^+^EMCN^+^ vessels (**f**). Quantitative analyses to show the length Fraction of CD31^+^EMCN^−^ arterial vessels (**g**). *n* = 3, *P < 0.05 by ANOVA. Col 1a (2.3) GFP^+^ cells as Cyan, SHG as white, CD31as red and EMCN as green. Scale bar = 100 μm

The marked difference in bone formation along superior and dura periosteum prompted us to further examine blood vessel density and vessel type distribution at the different site of parietal bone. To simultaneously examine osteogenesis and angiogenesis, we utilized *Col 1a (2.3) GFP* mouse model which labels osteoblasts with green fluorescence protein (GFP). To determine blood vessel types, samples were stained with Endomucin (EMCN) and CD31 antibodies which allowed us to differentiate arterial, venous, and vessels utilizing high-resolution MPLSM.^20–22^ As shown (Fig. 4a–c), prior to injury, vessel networks were distinctly different in morphology and density at superior and dura periosteum as well as in bone marrow space. The vessels at dura periosteum were better organized, comprising CD31^+^EMCN^+^ venous and capillary vessels as well as CD31^+^EMCN^−^ small arterial vessels, with large clusters of active Col 1a (2.3) GFP^+^ osteoblasts scattered within the vessel network (Fig. 4b). In comparison, vessel network at superior periosteum were thin and sparse with fewer and much smaller clusters of osteoblasts observed. (Fig. 4a). In contrast to the vessels at dura and superior periosteum, those in cortical marrow space primarily comprised of large diameter CD31^+^EMCN^+^ vessels with a few CD31^+^EMCN^−^ vessels running along the vascular channels in un-injured bone. Many of these CD31^+^EMCN^+^ vessels were found closely associated with GFP^+^ osteoblasts indicating they are type H vessels as reported in literature (Fig. 4c).^22,23^ Due to less uniform penetration of the antibodies in bone tissue, these vessels were at times showed unevenly staining of CD31 and EMCN. We therefore refrained from classifying the vessel types solely based on the fluorescence intensity of CD31 and EMCN staining. Quantitative analyses of the blood vessels at all three sites showed that the total vessel density (Vessel Volume Fraction), as indicated by CD31^+^ staining, was 5-fold higher in the dura periosteum than in the superior periosteum (Fig. 4d). Dura periosteum also harbored 10-fold more clusters of active osteoblasts along the surface of bone than superior periosteum (Fig. 4e, *P* < 0.05). Among these vessels, both CD31^+^EMCN^+^ and CD31^+^EMCN^-^ vessels were found at the highest density in the dura periosteum. The superior periosteum had the lowest volume fraction of all types of vessels, as well as the least number of osteoblasts. The percentage of CD31^+^EMCN^+^ vessels relative to the total CD31^+^ vessels by volume was (85 ± 13)% in the superior periosteum and (91 ± 2)% in the dura periosteum. The percentage of CD31^+^EMCN^−^ vessels relative to total CD31^+^ vessels by volume was (8.1 ± 5)% in the superior periosteum and (9.4 ± 0.9)% in the dura periosteum. While the vessel density was significantly higher in the dura periosteum compared to the superior periosteum, the ratio, and composition of CD31^+^EMCN^+^ and CD31^+^EMCN^−^ vessels appeared to be similar at both sites. In contrast, predominant vessel type in marrow space was CD31^+^EMCN^+^ vessels, with less than 1% of the vessels identified as CD31^+^EMCN^−^.

**Fig. 5:**
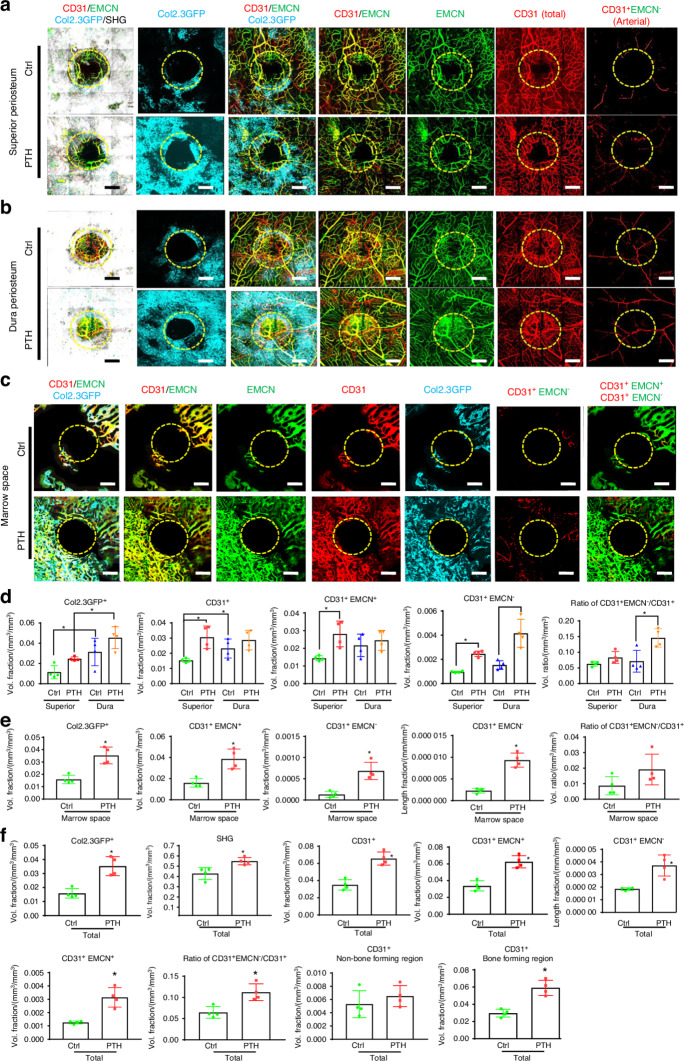
Osteoblasts and vessel subtypes in superior periosteum, dura periosteum, and in cortical bone marrow space during defect repair with or without rhPTH treatment. Representative MPLSM images of vessel network around bone defects in superior periosteum (**a**), dura periosteum (**b**), and bone marrow space (**c**) at 21 days following surgery. **d** Quantitative analyses to compare microvasculature in superior vs. dura periosteum with or without rhPTH treatment, illustrating the volume fractions of Col 1a (2.3) GFP^+^ osteoblasts, the total CD31^+^ vessels, CD31^+^EMCN^+^, CD31^+^EMCN^−^ arterial vessels, and the arterial vessel volume ratio over total vessel volume. **e** Quantitative analyses of microvasculature in marrow space with or without rhPTH treatment, illustrating the volume fractions of Col 1a (2.3) GFP^+^ osteoblasts, the total CD31^+^ vessels, CD31^+^EMCN^+^, CD31^+^EMCN^-^ arterial vessels, and the arterial vessel volume ratio over total vessel volume. The length fraction of CD31^+^EMCN^-^ arterial vessels is also shown. **f** Quantitative analyses of the overall microvasculature in defects treated with or without rhPTH, illustrating the volume fractions of Col 1a (2.3) GFP^+^ osteoblasts, the total CD31^+^ vessels, CD31^+^EMCN^+^, CD31^+^EMCN^−^ arterial vessels, the length fraction of CD31^+^EMCN^−^ arterial vessels, and the arterial vessel volume ratio over total vessel volume. The total CD31^+^ vessels in bone-forming and non-bone-forming regions are also illustrated. *n* = 4, *P < 0.05 by ANOVA. Circles outline the region of the defects. Col 1a (2.3) GFP^+^ cells as Cyan, SHG as white, CD31 as red, and EMCN as green. Isolated CD31^+^EMCN^+^ and CD31^+^EMCN^−^ vessels are shown as green and red as indicated. Scale bar = 200 μm

Osteogenesis and angiogenesis were further examined following injury in Col 1a (2.3) GFP mice with or without treatment with rhPTH. Vessel types and their distribution at the superior and dura periosteum, as well as in marrow space were analyzed at weeks 3 following injury. As shown, vessel densities were enhanced at all three sites (Fig. 5a–c), with marked induction of Col 1a (2.3) GFP^+^ cells following intermittent treatment of rhPTH. When separately evaluating the healing at the superior and dura periosteum, we found that intermittent rhPTH differentially regulated the vessel types at both superior and dura periosteum at week 3 (Fig. 5a, b). The rhPTH treatment also elicited a strong induction of the large diameter CD31^+^EMCN^+^ type H vessels within bone tissue (Fig. 5c). Quantitative MPLSM analyses (Fig. 5d), showed that dura periosteum had 2.8-fold more Col 1a (2.3) GFP^+^ osteoblasts than superior periosteum (*P* < 0.05), regardless of rhPTH treatment. Dura periosteum also had slightly more vessels than superior periosteum at day 21 post-op (*P* < 0.05). However, superior periosteum was more responsive to rhPTH than dura periosteum. Treatment of rhPTH strongly induced arterial vessel formation by about 2.8-fold at both superior and dura periosteal surfaces (*P* < 0.05), enhancing the volume ratio of arterial vessels over total vessel at dura periosteum (*P* < 0.05). This result suggested that rhPTH promoted the perfusion of oxygen and nutrients via increasing arterial vessels in the healing tissue. Treatment of rhPTH also had an approximately 2.5-fold increase of the large diameter CD31^+^EMCN^+^ vessels in bone marrow space (Fig. 5e, *P* < 0.05). The induction was associated with a 5-fold increase in small CD31^+^EMCN^−^ arterial vessels in bone tissue (*P* < 0.05).

Overall angiogenesis and neovascularization were evaluated combining all three sites at weeks 3 post-surgery (Fig. 5f). As shown, rhPTH markedly increased bone formation as evidenced by a 33% induction of SHG (*P* < 0.05) and a 2.3-fold increase in Col 1a (2.3) GFP^+^ cells (*P* < 0.05). The rhPTH had a strong effect on all three types of vessels (CD31 total, CD31^+^EMCN^+^, and CD31^+^EMCN^−^) (*P* < 0.05), enhancing the volume ratio of arterial vessels over total vessels by 1.7-fold (*P* < 0.05). When separately evaluating the vessels in bone-forming and non-bone-forming regions, the induction of vessel formation was only observed in bone-forming regions associated with SHG or Col 1a (2.3) GFP^+^ cells (*P* < 0.05). PTH had no discernible effects on vessels in non-bone-forming regions, suggesting that its effects on vessel formation could be mediated indirectly.

### Minimal impact on bone healing following deletion of VHL in endothelial cells

**Fig. 6:**
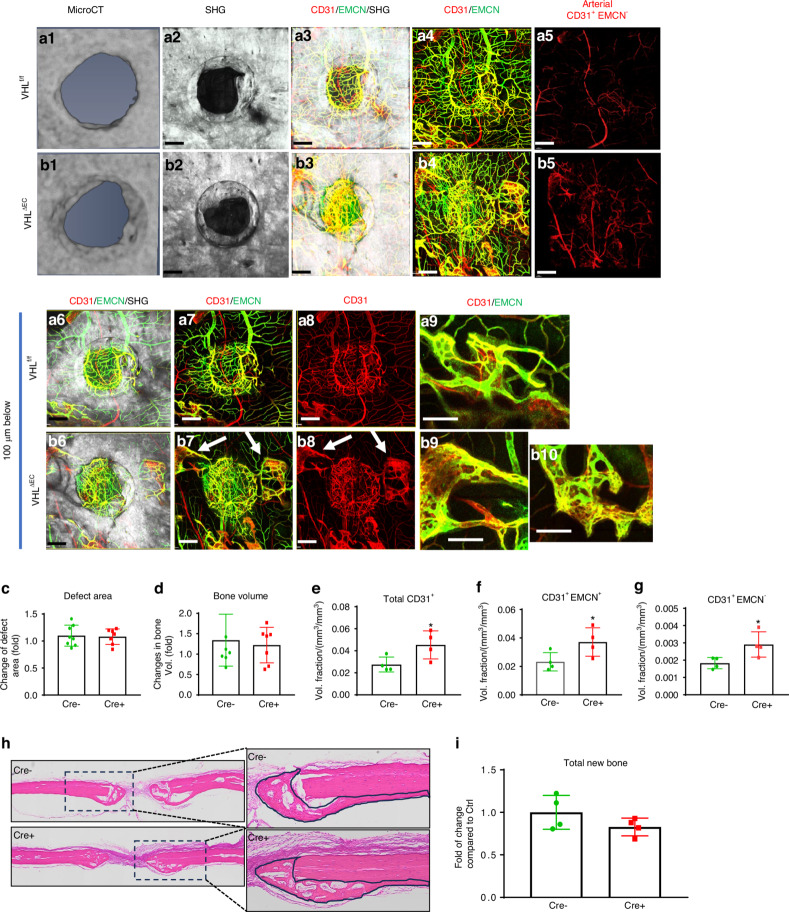
Conditional deletion of *VHL* in ECs had no appreciable effects on defect healing. Representative images of MicroCT (a1&b1) and MPLSM images (a2-9&b2-10) of the vessel networks around bone defect scanned from dura periosteum. Panel **a** are images obtained from control *VHL*^*f/f*^ mice and panel **b** are images from *VHL*^*ΔEC*^ mice. Images (a6-9&b6-10) are reconstructed at 100 μm below surface to show the vessel networks in bone tissue. Quantitative MicroCT analyses to compare bone formation (**c**) and defect area (**d**) in *VHL*^*f/f*^ and *VHL*^*ΔEC*^ mice. Quantitative MPLSM analyses to illustrate the volume fractions of the total CD31^+^ vessels (**e**), CD31^+^EMCN^+^ (**f**), CD31^+^EMCN^−^ arterial vessels (**g**). Col 1a (2.3) GFP^+^ cells as Cyan, SHG as white, CD31as red and EMCN as green. Isolated CD31^+^EMCN^+^ and CD31^+^EMCN^−^ vessels are shown as green and red as indicated. Scale bar = 200 μm. *n* = 4, *P < 0.05 by ANOVA. Histologic H&E staining of the defects (**h**) from *VHL*^*f/f*^ and *VHL*^*ΔEC*^ mice outlining the area of new bone at the repair site. Quantitative histomorphometric analyses (**i**) to show changes of total new bone formation in *VHL*^*f/f*^ and *VHL*^*ΔEC*^ mice. *n* = 4, *P* > 0.05 by Student’s *t-*test

The hypoxia inducible factor 1 (HIF1) pathway has a profound effect on bone formation and angiogenesis. Previous studies have shown that modifying the HIF1 pathway in endothelial cells (ECs) during bone development can affect bone formation by modulating the formation of different vessel types.^24^ To determine the potential impact of vessel type and density on cranial bone defect repair, the *Von Hippel-Lindau (VHL)* gene, which controls the ubiquitination and degradation of HIF1, was conditionally deleted at the onset of healing in EC via *Cdh5CreER*^*T2*^ mouse model. The deletion efficiency was confirmed in FACS-sorted ECs and by crossing *Cdh5CreER*^*T2*^ with Ai9 reporter mice (Fig. S2). Following the deletion of *VHL* gene in ECs, a small number of mice died within 3 weeks. The remaining survival mice were analyzed to determine bone formation and neovascularization 21 days post-surgery. Due to opulent bone formation on dura site compared to superior periosteal site, we scanned the cranial samples from dura periosteum for MPLSM analyses (Figs. 6a2-9, b2-10). As shown, deletion of *VHL* in EC led to increased vascularity of all vessel types at day 21 (*P* < 0.05). Compared to the controls, vessel density was increased at the surface (Figs. 6a4 vs. 6b4), associated with the appearance of the abnormal CD31^+^EMCN^+^ vessels in bone marrow space (Figs. 6b7-10, arrows). Quantitative analyses showed significantly increased volume fractions of all types of vessels around bone defects (Fig. 6e–g, *P* < 0.05). However, despite altered vessel density, deletion of *VHL* had no appreciable effects on bone formation and defect healing as indicated by MicroCT analyses (Figs. 6a1, b1, Fig. S3). Quantitative analyses showed no significant difference in bone formation or defect area between the control and *VHL*^*ΔEC*^ mice at days 21 post-surgery (Fig. 6c, d, *P* > 0.05), suggesting that the deletion of *VHL* in EC via *Cdh5CreER*^*T2*^ mouse model at the onset of healing was insufficient to significantly impact bone defect repair. The results were further confirmed by histologic and histomorphometric analyses comparing new bone at the site of repair (Fig. 6h, i, *P* > 0.05).

## Discussion

The calvarium has a dual embryonic origin from both neural crest and paraxial mesoderm.^25–27^ While the impact of different progenitor cells derived from neural crest or mesoderm on postnatal bone defect repair remain unclear, work from Quarto et al. suggests that the calvarial defect created at the frontal bone heals more rapidly than the defect created at the parietal bone, with the neural crest-derived frontal bone exhibiting greater osteogenic potential than the mesodermal-derived parietal bone.^28^ By comparing the bone regeneration capacities of human mesenchymal stem cells (MSCs) derived from neural crest and bone marrow, Srinivasan et al. show that neural crest-derived MSCs are more proliferative than bone marrow-derived MSCs in vitro. The neural crest MSC-seeded constructs exhibit more new bone formation than those seeded with bone marrow MSCs in the defect area.^29^ In our current study, we show that new bone formation along the dura periosteum is three times greater than that along the superior periosteum during bone defect repair. Using *Col 1a (2.3) GFP* mouse model, we further demonstrate a significantly higher number of osteoblasts at the dura periosteum than superior periosteum before and after injury, irrespective of intermittent rhPTH treatment. Utilizing *SMA-creER*^*T2*^ mouse model known to target periosteum progenitors,^30^ we show that SMA-CreER^T2^ labels superior periosteum of the parietal bone but not dura periosteum. Selective deletion of *PTH1R* via *SMA-CreER*^*T2*^ results in a significant reduction of new bone in superior but not dura periosteum following rhPTH treatment. In contrast, deletion of *PTH1R* in mature osteoblasts via *Col 1a (2.3)-CreER*^*T2*^ mouse model reduces new bone formation at both superior and dura periosteum. Taken together, these data strongly suggest that superior and dura periosteum of mouse calvarial bone harbor distinct adult progenitor cell pools exhibiting different osteogenic potential during repair. The difference in their osteogenic potential may stem from the interplay of genes and signaling pathways defining the distinct embryonic origin of the neural crest and mesoderm-derived calvarial cells. To further support this notion, a recent study using single-cell transcriptomic analyses to map developing meninges fibroblasts reveal dura fibroblast differs with other meningeal fibroblast and has distinct transcriptional signatures. It shows enriched expression of *Crabp2* and *Ogn*, but unique expression of *Fxyd5*, *Dkk2*, *Tgfbi*, and *Mgp*.^31^

Besides the difference in osteogenic progenitor cells, our study further demonstrates distinct blood vessel networks associated with superior and dura periosteum before and after injury. The blood vessel network of the entire murine calvarium has previously been analyzed via light sheet microscopy.^32^ While light sheet microscopy enables efficient scanning of large areas of skull bone, further clarification is needed to resolve the details of the vessel networks associated with the superior and dura periosteum at a higher resolution. The MPLSM allows analyses of blood vessel network based on the intrinsic SHG signals derived from bone tissue, permitting more precise segmentation of the blood vessel networks at the defined location of the calvarial bone. By separately evaluating the microvasculature in the superior and dura periosteum, our analyses show a 5-fold higher density and a better organized arterial-venous vascular network associated with abundant osteoblast clusters in dura periosteum compared to superior periosteum prior to injury. Following injury, a greater increase in vessel formation in superior periosteum than dura at day 21 post-op was recorded following rhPTH treatment, likely due to the low density of vessels and a delayed response to injury at the onset of healing.^20^ While intermittent treatment of rhPTH does not increase the overall vascular density in dura periosteum at day 21 post-surgery, anabolic rhPTH significantly enhances arterial vessel volume/length as well as the arterial vessel ratio at dura periosteum, suggesting an important role of tissue oxygenation in rhPTH-enhanced repair. Taken together, our current study demonstrates the unique properties and the potential underlying mechanisms contributing to the heightened osteogenic potential of the dura periosteum during calvarial bone defect repair.

The differential osteogenic activity and microvessel distribution in superior and dura periosteum have strong indications in bone tissue engineering applications for cranial bone defect repair. In a study utilizing 3D-printed bio-scaffolds, a better bone formation has been noted in the tissue-engineered materials facing the side of dura.^33^ By placing a barrier membrane on the periosteum or dura to block cell migration and macromolecular protein diffusion, Gosain et al. show that barrier membrane placed at the periosteum site fails to prevent bone repair whereas barrier membrane placed at the dura site effectively blocks the defect repair.^34^ Insertion of a semipermeable or nonpermeable membrane between dura and human adipose-derived stromal cells (hASCs) can disrupt the direct contribution of dural progenitor cells as well as paracrine signals from the dura mater.^35^ These studies are consistent with the observations in our current study, highlighting the unique property of dura periosteum as a major reservoir for osteogenic and angiogenic cells. Future studies are needed to further characterize this unique pool of osteogenic mesenchymal progenitor cells on skull bone.^36^

Studies aimed at defining organotypic vessels in bone have established type H vessels as the specialized vessel type coupling to osteoblasts for bone formation during development and repair.^22^ As described, type H vessels directly connect to an arterial vessel in bone, suggesting that these vessels are better oxygenated.^37^ In our current study, we find that intermittent rhPTH markedly enhances type H vessel formation in cortical bone marrow space during healing. These CD31^+^EMCN^+^ large diameter type H vessels are tightly associated with osteoblasts and distinct from the vessel network in superior and dura periosteum. The expansion of these vessels likely requires coordinately activities of osteoblasts, osteoclasts, and endothelial cells,^38,39^ as marrow spaces within and around the newly formed bone are significantly expanded upon rhPTH treatment. In addition to type H vessels, the arterial vessel ratio is also significantly increased upon treatment of rhPTH. This result is consistent with our previous study which shows an increased arterial vessel ratio during BMSC-mediated cranial defect repair regeneration.^21^ The induction of the arterial vessels during repair underscores the importance of maintaining the perfusion of the repair region to sustain tissue oxygenation. While osteoblasts are shown to prefer a hypoxic microenvironment, adequate oxygen and nutrient supply are required to promote osteoblastic function and bone formation. In addition to osteoblasts, osteoclasts which reportedly utilize oxidative phosphorylation (OXPHOS),^40,41^ are also involved in remodeling of transcortical channels and marrow space in newly formed bone. Thus, a well-organized blood vessel network, capable of adapting to the specific requirements of the multiple cell types in a localized microenvironment, is crucial for repair and reconstruction of bone defects.

It is interesting to note that the significant effects of rhPTH on the formation of microvessels are solely observed at the bone-forming regions, rather than the non-bone-forming region, suggesting that the effects of PTH on vessel formation are indirect. This observation motivates us to examine whether targeting blood vessel alone could lead to improved bone healing. To this end, we employ a well-established VHL floxed conditional knockout mouse model, known to result in gain-of-function of HIF1 pathway upon gene deletion. Manipulation of HIF1 pathway, including deletion of *VHL* in EC, has a profound effect on angiogenesis and further affects the arterial vessel specification during embryonic development.^42–45^ Manipulation of the HIF pathway in bone-forming cells has also demonstrated a profound effect on bone formation during development and repair.^46,47^ Targeted deletion of *VHL* gene in ECs impacts type H vessel development and subsequent bone formation in neonatal mice.^24^ To determine whether enhancing vessel formation by manipulation of HIF pathway could impact bone repair, we delete the *VHL* gene via *Cdh5-CreER*, which is known to target all ECs.^48^ To our surprise, deletion of *VHL* in ECs has no significant impact on bone repair and regeneration in spontaneous cranial defect repair, as evidenced by MicroCT and histomorphometric analyses. While we observe an enhanced vessel formation, many vessels appear to be abnormal in cortical marrow space, having little impact on bone healing. These data suggest that promoting blood vessel formation alone may not be sufficient to induce bone formation. Effective repair and reconstruction of bone tissue requires coordinated actions from osteoblasts, osteoclasts, endothelial cells, and potentially other cell types to achieve optimal outcomes.

## Conclusion

Our current study demonstrates a differential response that depends on an organized angiogenesis coupling to osteoblasts at superior and dura periosteum during calvarial bone defect repair. The study offers new insights into the mechanisms of healing, which could inform strategies for directing repair and tissue engineering of bone tissue at the site of cranial bone defects.

## Materials and methods

### Mouse strains and animal care

The *Col 1a (2.3) GFP* transgenic mice, which specifically label mature osteoblasts with GFP, were originally obtained from Jackson Laboratory.^49,50^ *SMA-CreER*^*T2*^ mouse model^51^ previously shown targeting long bone periosteum was obtained from Dr. Ivo Kalajzic at Uconn Health. *Col 1a (2.3)-CreER*^*T2*^^52^ (Cat#016241) and *VHL*^*f/f*^ mouse model^53^ were obtained from Jackson Laboratory. *PTH1R*^*f/f*^ mice^54^ were kindly provided by Dr. Henry Kronenberg at Havard Medical School. *Cdh5-CreER*^*T2*^ mice^48^ were obtained through Dr. Ralf Adams via Material Transfer Agreement. Gt (ROSA)26Sortm9(CAG-tdTomato) known as Ai9 mice were purchased from Jackson Laboratory. Genomic DNA preparations from mouse ear snips were used to determine the genotypes of each mouse strain via polymerase chain reaction (PCR). All in vivo experiments were performed using adult 8–12-week-old animals housed in pathogen-free, temperature and humidity-controlled facilities with a 12 h day-night cycle in the vivarium at the University of Rochester Medical Center. All cages contained wood shavings, bedding, and a cardboard tube for environmental enrichment. All experimental procedures were reviewed and approved by the University Committee on Animal Resources. General anesthesia and analgesia procedures were performed based on the mouse formulary provided by the University Committee on Animal Resources at the University of Rochester. The animals’ health status was monitored throughout the experiments by experienced veterinarians according to the Guide for the Care and Use of Laboratory Animals outlined by the National Institute of Health.

Tamoxifen (Sigma, T5648) was dissolved in corn oil (20 mg/mL) and administered by i.p. injection at day 1 and 2 post-surgery (0.10–0.15 mg Tamoxifen per gram mouse body weight) for lineage tracing. Tamoxifen was given at intraperitoneal injection to every mouse, including control and experimental groups, at a dose of 1 mg/mice at 5 different time point, including 2 days prior to surgery, and 2, 4, 6, 14 days after surgery for gene deletion experiments. Teriparatide rhPTH^6,55^ was a gift from Eli Lily and administrated daily at a dose of 80 μg/kg/d via Sub-Q as previously described.

### Animal surgery and cranial bone defect model in mice

Procedures for creating a cranial defect have been previously described.^56,57^ Briefly, the mouse under anesthesia via i.p. injection of Ketamine/xylazine mixture (100 mg/kg ketamine and 10 mg/kg xylazine) had its hair removed from surgical site of the skull. A stereotaxic instrument (Stoelting Inc., Wood Dale, IL) was used to stabilize the mouse head for surgery under a dissection microscope. A 1 mm in diameter full-thickness defect was created in the parietal bone of mouse calvarium using a same-sized Busch inverted cone bur (Armstrong Tool & Supply Company, Livonia, MI). Great care was taken to minimize damage to the surrounding periosteum during and after surgery. The bone wounds were closed via suture and the healing was monitored over a period of 3–5 weeks. All samples were harvested for MicroCT, MPLSM imaging, and histologic analyses at the indicated time points.

Longitudinal evaluation of cranial defect repair via MicroCT. The healing dynamics of a 1 mm-sized cranial defect repair was examined by a Scanco VivaCT 40 system (Scanco Medical AG, Bassersdorf, Switzerland) at weeks 1, 3 and 5 after surgery. These imaging data were anonymized and exported as DICOM files for the evaluation of graft bone formation and host bone response using Amira software version 6.0 (FEI Visualization Sciences Group, Hillsboro, OR). DICOM files from Micro-CT scanning were imported into Amira. The 1 mm defect region and the surrounding region of a 0.5 mm radius were drawn using VolumeEdit in Amira. The region of interest was defined as 90 * 90 * 60 pixels. The bone volumes within the defect and the regions immediately adjacent to the defect were evaluated as previously described.^21^ To determine the defect area, images of maximum projection were used to trace the area of the defect and subsequently used to compare the defect size over time in each sample. The percent change in bone volume and defect area at weeks 3 and 5 compared to week 1 were utilized for evaluation of defect healing.

#### Histologic and histomorphometric analyses of defect healing

Mice were perfused with 4% paraformaldehyde (Electron Microscopy Sciences, Hatfield, PA, USA) following surgery as previously described.^21^ The cranial bone was decalcified with 14% EDTA for 5 days and washed with PBS. Mid-sagittal frozen sections (10 μm thick) were prepared via cryosection and stained with Hematoxylin & Eosin (H&E). High resolution digital images of the histologic sections were obtained via Olympus VS110™ Virtual Slide Scanning System (Olympus, Tokyo, Japan). To evaluate bone defect healing, the newly formed bone area was traced and outlined manually in Image J (National Institutes of Health, Bethesda, Maryland, USA), based on the distinctive H&E staining, collagen alignment, and bone cement line/demarcation that separate new bone and old bone at the leading edge of the defect, superior and dura periosteum (as shown in Fig. S1). The percent changes in area of new bone compared to the controls were calculated and plotted.

To determine the targeting efficiency and targeting cell population in *SMA-CreER*^*T2*^ and *Col 1a (2.3)-CreER*^*T2*^ mice, two consecutive Tamoxifen (TM) i.p. injections (20 mg/kg) were performed on day 1 and 2 post-surgery. Samples were harvested at weeks 3 and 5. Cryosections were prepared for fluorescence microscopy as described above. The cryosections were scanned and imaged via Olympus VS110™ Virtual Slide Scanning System to illustrate the location of the tdTomato-positive cells at the defect site.

Immunofluorescent staining of blood vessels and multiphoton laser scanning microscopy (MPLSM). Cryosections were blocked with 3% BSA (prepared with PBSTX, 1% triton X-100 in PBS) for 24 h. The samples were treated with 3% bovine albumin in PBS containing 0.3% Triton X-100 and then stained with CD31 antibody (1:100 dilution, Biolegend, San Diego, CA) and EMCN antibody (1:50 dilution, Santa Cruz Biotechnology, Santa Cruz, CA). To enhance staining and antibody penetration, samples were centrifuged with staining solution at 4 °C, 600 g for 2 h, then incubated at 4 °C for 5 more days at 4 °C. Following wash with PBSTX and PBS, samples were mounted with Mowiol on glass slides (prepared follow Cold Spring Harbor Protocols http://cshprotocols.cshlp.org/content/2006/1/pdb.rec10255). The samples were imaged via MPLSM as described.^21^ Briefly, an Olympus FVMPE-RS system equipped with two lasers: Spectra-Physics InSightX3 (680–1 300 nm) and Spectra-Physics MaiTai DeepSee Ti:Sapphire laser (690–1 040 nm), and 25X water objective (XLPLN25XWMP2, 1.05NA) was used for high-resolution imaging. Images were acquired at 512 × 512 pixels using resonant scanners with the laser tuned to 780 nm. The fluorescence of GFP, RFP, far-red RFP, and Second Harmonic Generation (SHG) signals were collected with a 517/23 nm, a 605/25 nm, a 665/20 nm, and a 390/20 nm bandpass filters (Semrock), respectively. The 2D slice viewing and 3D reconstruction of the defect were performed using a combination of Imaris (Bitplane Inc., Concord, MA) and Amira image analysis software (Visage Imaging, Berlin, Germany).

### Quantitative and histomorphometric analyses of neovascularization at the site of cranial bone defect repair

All samples were scanned from superior and dura side of the cranial bone for analyses. A detailed method for quantitative analyses of blood vessels at the site of cranial defect repair has been previously described.^56,57^ Briefly, CD31^+^EMCN^−^ or CD31^+^EMCN^+^ vessels along with SHG and Col 1a (2.3) GFP cells were reconstructed in a 3D format using a multichannel z-series stack. To analyze spontaneous healing in 1 mm defect, a 3-dimensional cubic region of 2 × 2 mm (16 tiles of 512 × 512 z-series stack) up to 200 μm in depth, comprised of the defect and the surrounding area was created as the region of interest (ROI) for quantitative analyses. The vascular network within ROI was subsequently isolated using Amira Segmentation Editor to obtain the final segmented vascular image. Using Amira’s AutoSkeleton module, which implements a distance-ordered homotopic thinning operation, the segmented 3D vascular network was further skeletonized to generate a line-based network that was topologically equivalent to the original network. The skeleton was superimposed on the original image to assess the relative accuracy of this method. The final skeletonized vessel network was obtained by manually retracing of the skeletons using Amira’s Filamental Editor to remove false segments. Based on the skeletonized network, vessel length fraction (L.Fract.) (i.e., ratio of vessel length to total volume) was read from the Amira software. Quantitative and histomorphometric analyses of neovasculature as described above were performed simultaneously with volumetric quantification of Col 1a (2.3) GFP cells and SHG using Amira segmentation Editor and volumetric analyses protocol. Analyses were performed in a group of 4 mice, covering the entire defect regions. Analyses and evaluation of the volumes of various types of vessels in bone-forming and non-bone-forming regions were conducted using combination of Amira and Image J as previously described.^56,57^ The bone-forming and non-bone-forming regions were defined and contoured for analyses based on Col 1a (2.3) GFP^+^ cells as well as the SHG signals from bone tissue.

### Statistical analyses

All data were presented as mean ± standard deviation (S.D.) Statistical analyses were conducted via GraphPad Prism (GraphPad Prism, San Diego, CA). A *t*-test or an ordinary one-way ANOVA, with a Tukey Post-Hoc, was utilized on normally distributed data. In all analyses, a *P*-value of <0.05 was considered statistically significant. The Student’s *t*-test was used to compare the means when only two groups of samples were included in the experiments. The statistical significance level was set at *P < 0.05, **P < 0.01, ***P < 0.001.

## Supplementary information

Supplemental Data
