# Supplementary material for: Differential bone and vessel type formation at superior and dura periosteum during cranial bone defect repair
Source: Bone Res. 2025 Jan 13;13:8. doi: 10.1038/s41413-024-00379-9 (PMC11729862; doi:10.1038/s41413-024-00379-9)
Supplement: Supplementary file 1 — Supplemental Data [file 41413_2024_379_MOESM1_ESM.pdf]

Supplemental Fig. S1

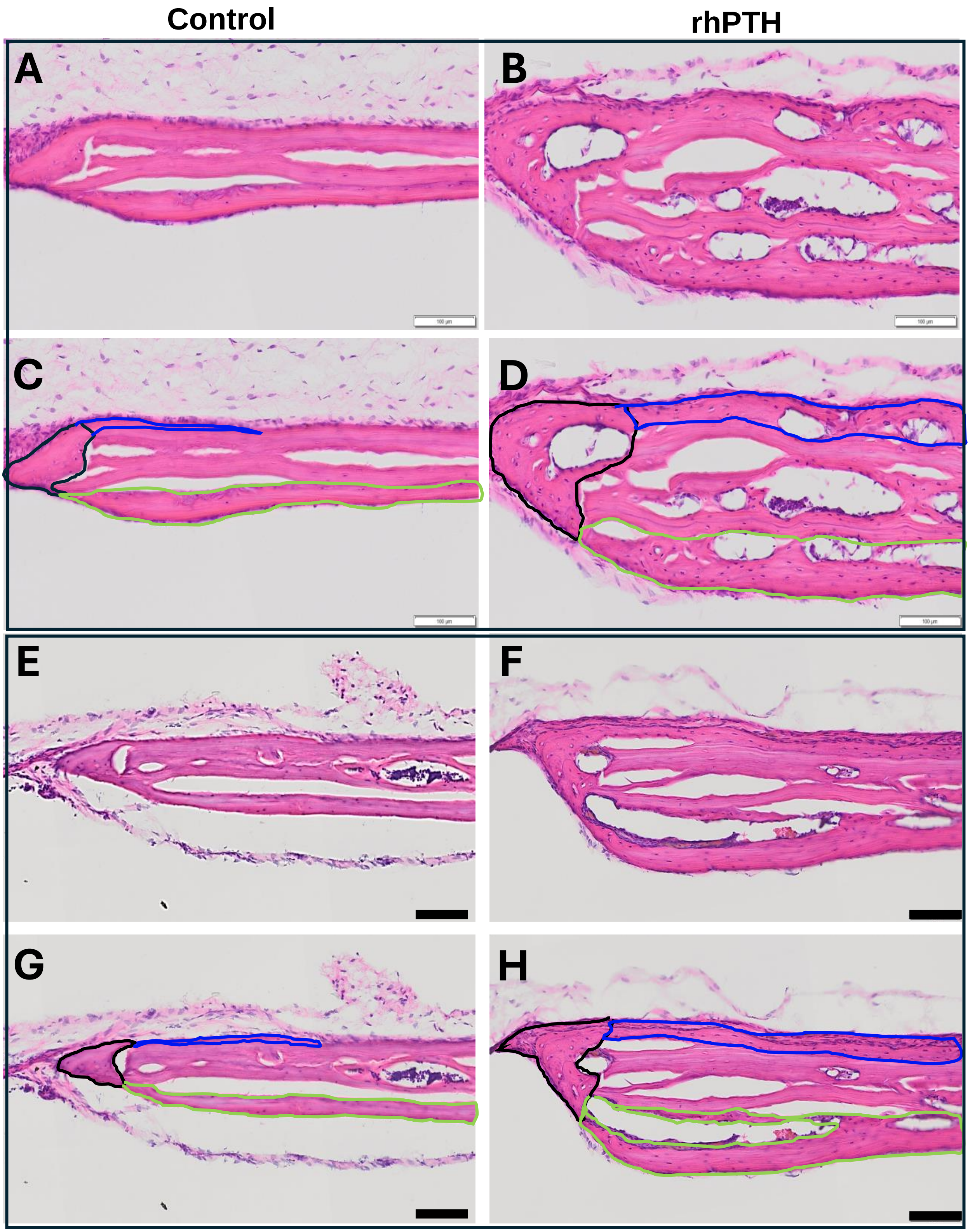

Suppl. Fig. S1. Histomorphometric analyses to illustrate new bone formation at superior periosteum, dura periosteum and the leading edge of bone defect. (A-B & E-F) H&E staining of the cranial defect sections in control and rhPTH treated mice. Noted the demarcation line between the new bone and old bone on the tissue sections in control and PTH treated mice. (C-D & G-H) New bone formed at the superior, dura periosteum, and the leading edge were outlined in different color. Superior periosteum (blue), leading edge (black), dura periosteum (green).

Supplemental Fig. S2

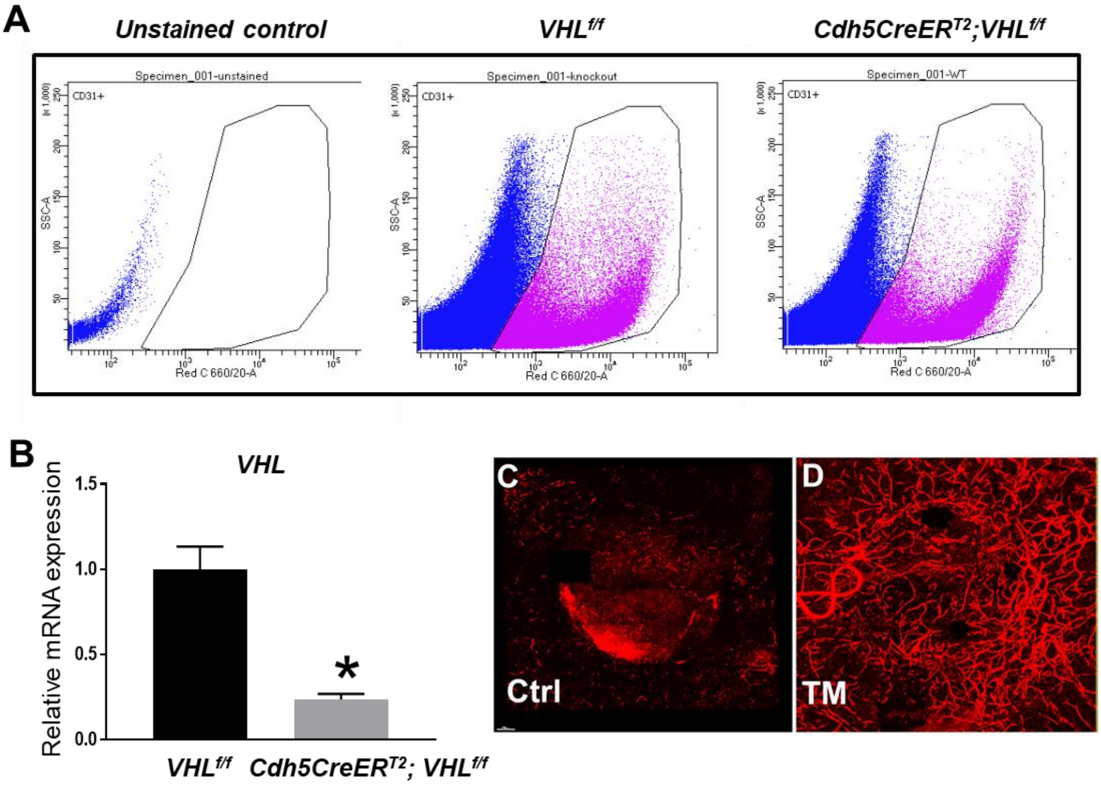

Suppl. Fig. S2. Cells were isolated from lung of *VHL$^{f/f}$* *and Cdh5CreER^{T2};VHL$^{f/f}$*mice via collagenase D digestion. Isolated cells were filtered and stained with CD31 antibody conjugated with Alexa 647. CD31^{+} cells were gated based upon unstained negative control and collected via FACS sorting (A). Expression of *VHL* gene were determined by RT-PCR following normalization with *GAPDH*. Tamoxifen (TM) treatment induced strong Cdh5CreER^{T2} recombination in EC at the cranial defect site as indicated by Cdh5CreER^{T2}; Ai9 mice (C&D).

Supplemental Fig. S3.

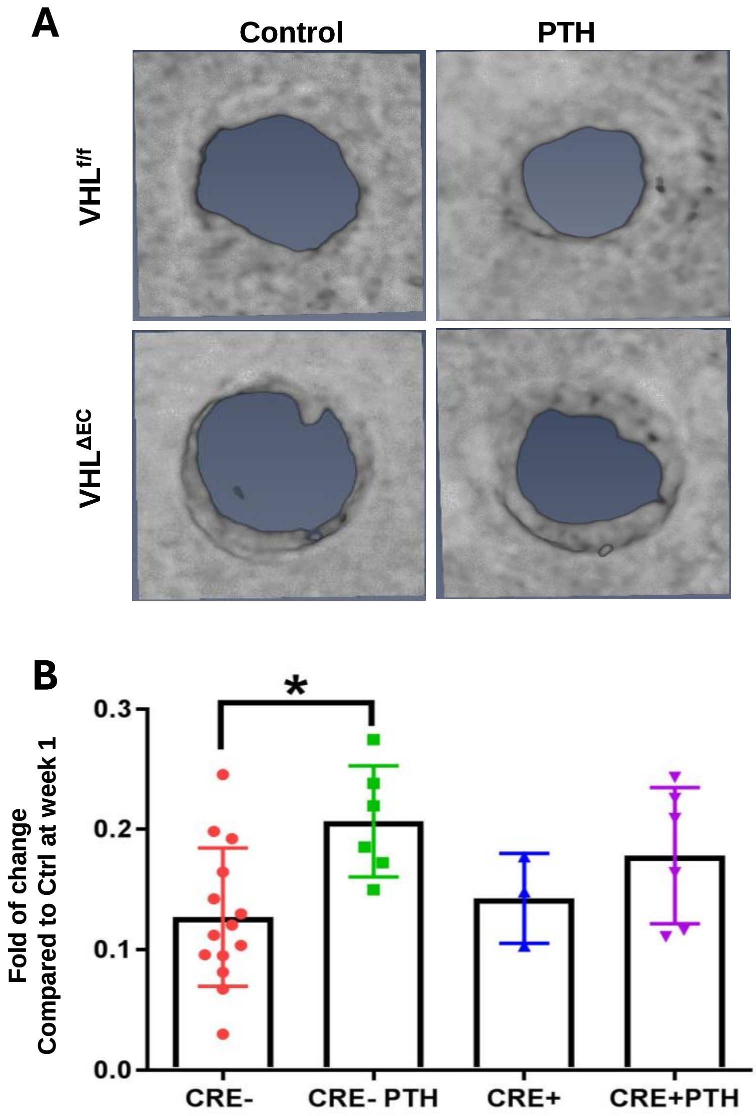

Suppl. Fig. S3. Conditional deletion of *VHL* in ECs had minimal effects on bone defect repair even following PTH treatment for 18 days. (A) Representative images of MicroCT in $VHL^{f/f}$ and $VHL^{\Delta EC}$ mice after at least 18 days of treatment. (B) Quantitative MicroCT analyses to compare bone formation show no significant changes of bone formation following deletion of VHL in ECs. *, n=5, p>0.05 by ANOVA analyses.
